# Multiple Cytochrome P450 Genes: Their Constitutive Overexpression and Permethrin Induction in Insecticide Resistant Mosquitoes, *Culex quinquefasciatus*

**DOI:** 10.1371/journal.pone.0023403

**Published:** 2011-08-12

**Authors:** Nannan Liu, Ting Li, William R. Reid, Ting Yang, Lee Zhang

**Affiliations:** 1 Department of Entomology and Plant Pathology, Auburn University, Auburn, Alabama, United States of America; 2 Genomics and Sequencing Laboratory, Auburn University, Auburn, Alabama, United States of America; New Mexico State University, United States of America

## Abstract

Four cytochrome P450 cDNAs, *CYP6AA7*, *CYP9J40*, *CYP9J34*, and *CYP9M10*, were isolated from mosquitoes, *Culex quinquefasciatus*. The P450 gene expression and induction by permethrin were compared for three different mosquito populations bearing different resistance phenotypes, ranging from susceptible (S-Lab), through intermediate (HAmCq^G0^, the field parental population) to highly resistant (HAmCq^G8^, the 8^th^ generation of permethrin selected offspring of HAmCq^G0^). A strong correlation was found for P450 gene expression with the levels of resistance and following permethrin selection at the larval stage of mosquitoes, with the highest expression levels identified in HAmCq^G8^, suggesting the importance of *CYP6AA7*, *CYP9J40*, *CYP9J34*, and *CYP9M10* in the permethrin resistance of larva mosquitoes. Only *CYP6AA7* showed a significant overexpression in HAmCq^G8^ adult mosquitoes. Other P450 genes had similar expression levels among the mosquito populations tested, suggesting different P450 genes may be involved in the response to insecticide pressure in different developmental stages. The expression of *CYP6AA7*, *CYP9J34*, and *CYP9M10* was further induced by permethrin in resistant mosquitoes. Taken together, these results indicate that multiple P450 genes are up-regulated in insecticide resistant mosquitoes through both constitutive overexpression and induction mechanisms, thus increasing the overall expression levels of P450 genes.

## Introduction

Cytochrome P450s have long been of particular interest because they are critical for the detoxification and/or activation of xenobiotics such as drugs, pesticides, plant toxins, chemical carcinogens and mutagens. They are also involved in metabolizing endogenous compounds such as hormones, fatty acids, and steroids. Basal and up-regulation of P450 gene expression can significantly affect the disposition of xenobiotics or endogenous compounds in the tissues of organisms and thus alter their pharmacological/toxicological effects [1]. Insect cytochrome P450s are known to play an important role in detoxifying exogenous compounds such as insecticides [2], [3] and plant toxins [4], [5]. A significant characteristic of insect P450s associated with the enhanced metabolic detoxification of insecticides is the increase in the levels of P450 proteins and P450 activity that results from constitutive overexpression of P450 genes in insecticide resistant insects, which has been implicated in the development of resistance to insecticides [3], [6]–[11] and tolerance to plant toxins [12], [13]. Another feature of some insect P450 genes is that their expression can be induced by both exogenous and endogenous compounds [3], a phenomenon known as induction. It has been suggested that the induction of P450s and their activities in insects is involved in the adaptation of insects to their environment and, hence, the development of insecticide resistance [14]–[16].

While all insects probably possess some capacity to detoxify insecticides and xenobiotics, the degree to which they can metabolize and detoxify these toxic chemicals is of considerable importance to their survival in a chemically unfriendly environment [15] and to the development of resistance. The constitutively increased expression and induction of P450s are both thought to arise in response to increased levels of detoxification of insecticides [11], [16]. It has been suggested that many chemical inducers act as substrates for P450s and that the induction or modulation of P450s by such substrates will, in turn, reduce the effects of the substrates by enhancing substrate metabolism [16], [17]. The modulation of gene expression may therefore reflect a compromise between the insect's need to conserve energy and its ability to adjust to a rapidly changing environment by enhancing the activity of the detoxification system only when a chemical stimulus occurs [18].

The primary goal of our study was to investigate whether insecticide resistant insects may be uniquely resistant to insecticides due to their ability to mount an adequate cellular response when challenged with insecticides by up-regulating the production of P450s, which may, in turn, significantly diminish the toxicological effects of the insecticides on these insects [1]. In a previous study we used a combination of subtractive hybridization and cDNA array techniques to identify several P450 EST sequences overexpressed in resistant mosquitoes, *Cx. quinquefasciatus* [19]. The current study was focused on isolating the full-length cDNA sequences of those P450 ESTs, characterizing the expression profiles of these P450 genes from the same mosquito populations of *Cx. quinquefasciatu*s bearing different phenotypes in response to permethrin (susceptible, intermediate and highly resistant, [19]), and determining the response of these P450 genes to permethrin treatment among the three mosquito populations. Four cytochrome P450 cDNAs, *CYP6AA7*, *CYP9J40*, *CYP9J34*, and *CYP9M10*, were isolated with the primers designed according to the P450 EST sequences, the expression of these P450 genes were characterized, and the possible role of these genes in insecticide resistance was discussed.

## Materials and Methods

### Mosquito strains

Three strains of mosquito *Cx. quinquefasciatus* were studied: HAmCq^G0^, a field resistant strain collected from Huntsville, Alabama, USA [20]; HAmCq^G8^, the 8^th^ generation of permethrin-selected HAmCq^G0^ offspring; and S-Lab, an insecticide susceptible strain provided by Dr. Laura Harrington (Cornell University). All the mosquitoes were reared at 25±2°C under a photoperiod of 12∶12 (L:D) h [21] and fed blood samples from horses (Large Animal Teaching Hospital, College of Veterinary Medicine, Auburn University).

### Permethrin Treatment

Preliminary dose range, time course, and P450 gene induction assays were performed on late 3^rd^ instar larvae using a range of concentrations (LC_10_, LC_50_ and LC_90_) and a time course of 12, 24, 48, and 72h. Results of the pilot experiment, in which the induction of P450s in both resistant HAmCq mosquito populations showed a clear concentration (LC_50_)- and time (24 h)-dependent response. Based on these preliminary results, two different permethrin treatment experiments were conducted: 1) ∼1000 late 3^rd^ instar larvae of each of the three *Culex* mosquito strains were treated with permethrin at their respective LC_50_ concentrations (0.007ppm, 0.07ppm, and 20ppm for the S-Lab, HAmCq^G0^, and HAmCq^G8^ strains, respectively) and the expression of the P450 genes were examined 12, 24, 48, and 72h after the permethrin treatment; and 2) mosquito strains were treated with their corresponding LC_10_, LC_50_ and LC_90_ concentrations of permethrin (Table 1) and the surviving mosquitoes were collected for RNA extraction 24 h after permethrin challenge. Control mosquitoes that had not received the permethrin treatment (treated with acetone alone) were collected at the same time points as their permethrin treated counterparts. The experiments were repeated three times.

**Table 1:** Permethrin treatment of the late 3^rd^ instar larvae of *Culex* mosquitoes.

		Permethrin Treatment*		
Strain	n^a^	LC_10_ Treatment^b^	LC_50_ Treatment^b^	LC_90_ Treatment^b^
S-Lab	∼1000	0.003 ppm	0.007 ppm	0.02 ppm
HAmCq^G0^	∼1000	0.02 ppm	0.07 ppm	0.2 ppm
HAmCq^G8^	∼1000	10 ppm	20 ppm	30 ppm

*Each treatment was repeated 3 times.

a The number of late 3^rd^ instar mosquito larvae used at the beginning of each permethrin treatment.

b The concentrations of permethrin for these mosquitoes have been identified previously (Xu et al. 2006, Li et al. 2010).

### RNA extraction, cDNA preparation, and the 3′ and 5′ race

The 4^th^ instar larvae and 2–3 day-old female adults (without blood feeding) of each mosquito population had their RNA extracted for each experiment (except the permethrin treatment experiment, in which late 3^rd^ instar larvae were used) using the acidic guanidine thiocyanate-phenol-chloroform method [7]. Messenger RNA (mRNA) was isolated with oligotex-dT suspension method (QIAGEN). Three replications were performed, each on a different day. Rapid amplification of 3′ and 5′cDNA ends (3′and 5′-RACE) was carried out using the Marathon^TM^ cDNA Amplification Kit (Clontech) [22]. The first strand cDNAs were synthesized with AMV reverse transcriptase using mosquito mRNAs as templates. The double strand cDNA was synthesized following the protocol described by the manufacturer (Clontech). Adaptors were ligated to both ends of the double strand cDNA as described by the manufacturer. The double strand cDNAs were amplified by PCR with the primers designed according to our previous EST sequences [19] and AP1 primer (based on the sequence of the adaptor). The PCR products were cloned into PCR^TM^ 2.1 Original TA cloning vector (Invitrogen) and sequenced. The full length of the P450 cDNAs was generated by RT-PCR (reverse transcription-mediated polymerase chain reaction) using specific primer pairs according to the 5′and 3′end sequences of the putative P450 genes. Cloning and sequence analyses of the P450 cDNA fragments were repeated at least three times with different preparations of mRNAs, and three TA clones from each replication were verified by sequencing.

### Quantitative Real-time PCR (qRT-PCR)

Total RNA samples (0.5 µg/sample) from larval and adult mosquitoes were reverse-transcribed using SuperScript II reverse transcriptase (Stratagene) in a total volume of 20 µl. The quantity of cDNAs was measured using a spectrophotometer prior to qRT-PCR. qRT-PCR was performed with the SYBR Green master mix Kit and ABI 7500 Real Time PCR system (Applied Biosystems). Each qRT-PCR reaction (25 µl final volume) contained 1x SYBR Green master mix, 1 µl of cDNA, and a P450 gene specific primer pair (designed according to each of the P450 gene sequences, Table 2) at a final concentration of 3–5 µM. Al samples, including A ‘no-template’ negative control, were performed in triplicate. The reaction cycle consisted of a melting step of 50°C for 2 min then 95°C for 10 min, followed by 40 cycles of 95°C for 15 sec and 60°C for 1 min. Specificity of the PCR reactions was assessed by a melting curve analysis for each PCR reaction using Dissociation Curves software [23]. Relative expression levels for P450 genes were calculated by the 2^−ΔΔCT^ method using SDS RQ software [24]. The 18S ribosome RNA gene, an endogenous control, was used to normalize the expression of target genes [11], [25]. Preliminary qRT-PCR experiments with the primer pair (Table 2) of 18S ribosome RNA gene designed according to the sequences of the 18S ribosome RNA gene had revealed that the 18S ribosome RNA gene expression remained constant among all 3 mosquito strains, so the 18S ribosome RNA gene was used for internal normalization in the qRT-PCR assays. Each experiment was repeated three times with different preparations of RNA samples. The statistical significance of the gene expressions was calculated using a Student's *t*-test for all 2-sample comparisons and a one-way analysis of variance (ANOVA) for multiple sample comparisons (SAS v9.1 software); a value of *P*≤0.05 was considered statistically significant.

**Table 2:** The primers used for qRT-PCR reaction.

Gene	Primer Name	Primer Sequence*
18S Ribosomal RNA	18S rRNA F	5′ CGCGGTAATTCCAGCTCCACTA 3′
	18S rRNA R	5′ GCATCAAGCGCCACCATATAGG 3′
CYP9M10	qRTP450-1CxF	5′ ATGCAGACCAAGTGCTTCCTGTAC 3′
	qRTP450-1CxR	5′ AACCCACTCAACGTATCCAGCGAA 3′
CYP9J40	qRTP450-23CxF	5′ ACCCGAATCCGGGCAAGTTTGAT 3′
	qRTP450-23CxR	5′ AACTCCAAACGGTAAATACGCCGC 3′
CYP6AA7	P4505959F	5′ ATGACGCTGATTCCCGAGACTGTT 3′
	P4505959R	5′ TTCATGGTCAAGGTCTCACCCGAA 3′
CYP9J34	P45010546F	5′ ATCCGATGTCGGTAAAGTGCAGGT 3′
	P45010546R	5′ TGTACCTCTGGGTTGATGGCAAGT 3′

*The primers were designed according to sequences of each of corresponding genes.

## Results

### P450 genes and their expression profiles in *Cx. quinquefasciatus*

Four full lengths of P450 cDNAs were isolated from *Cx. quinquefasciatus* with 3′ and 5′ RACE using the specific primers designed from our previous P450 EST sequences [19]. The full lengths of these P450 cDNA sequences were assigned the names *CYP6AA7*, *CYP9J40*, *CYP9J34*, and *CYP9M10* (accession numbers: JF501089, JF501091, JF501092, JF501093, respectively) by the P450 nomenclature committee. The putative protein sequences of CYP6AA7, CYP9J40, CYP9J34, and CYP9M10 deduced from the cDNA sequences shared 99, 97, 100, and 99% identity with *Cx. quinquefasciatus CPIJ005959*, *CPIJ010543*, *CPIJ010546*, and *CYP9M10*, respectively; apart from *CYP9M10* [26] none have yet been reported to be involved in insecticide resistance.

**Figure 1:**
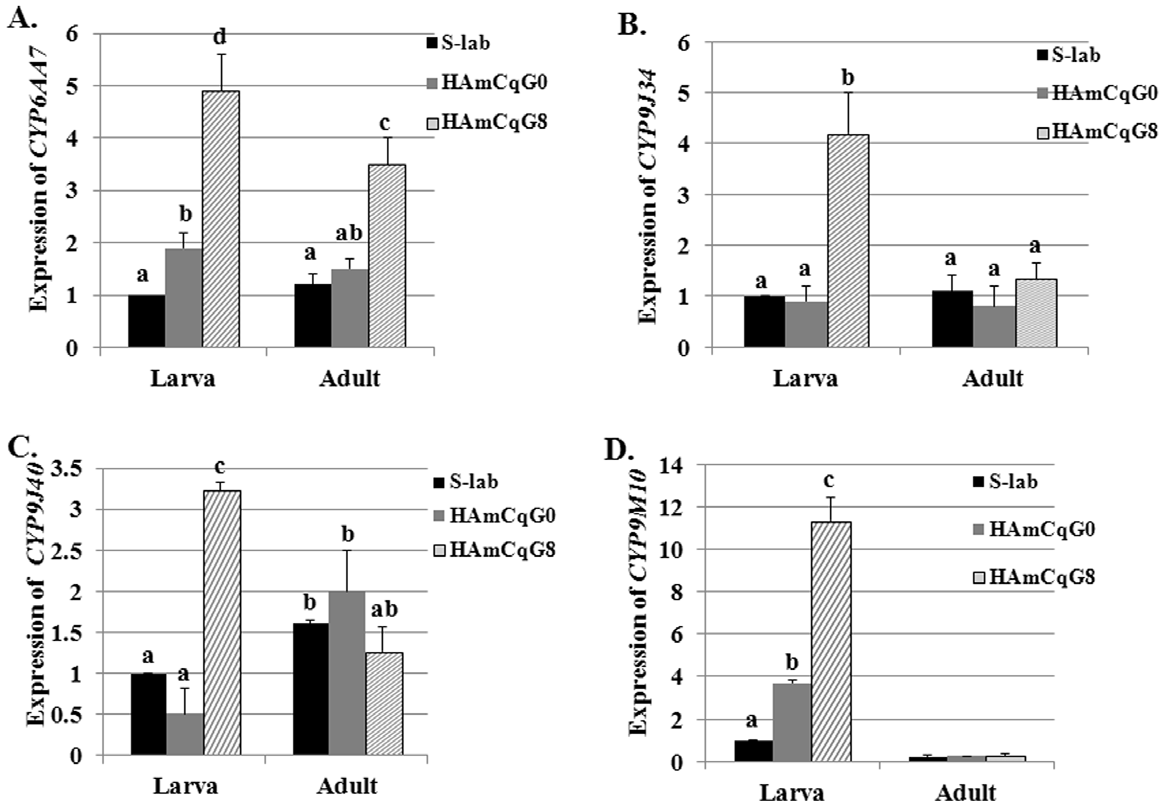
Expression analysis of *CYP6AA7*, *CYP9J34*, *CYP9J40*, and *CYP9M10* in mosquitoes, *Cx. quinquefasciatus*. The relative level of gene expression shown along the Y axis represents the ratio of the gene expression in each mosquito strain compared with that in the susceptible S-lab strain. The results are shown as the mean ± S.E. There was no significant difference (*P*≤0.05) in the levels of P450 gene expression among samples with the same alphabetic letter (i.e., a, b, or c). A. Relative *CYP6AA7* RNA levels. B. Relative *CYP9J34* RNA levels. C. Relative *CYP9J40* RNA levels. D. Relative *CYP9M10* RNA levels.

Diversity in the developmental expression and regulation of insect P450s is well established, so expression patterns of four P450 genes, *CYP6AA7*, *CYP9J34*, *CYP9J40* and *CYP9M10*, were examined in larval and adult mosquitoes of *Cx. quinquefasciatus*. Quantitative real-time PCR (qRT-PCR) analysis was performed to compare expression levels of the four P450 genes for larvae and adults among three different mosquito populations bearing different resistance phenotypes in response to permethrin, ranging from susceptible (S-Lab), through intermediate resistant (HAmCq^G0^, field parental population) to highly resistant (HAmCq^G8^ 8^th^ generation permethrin selected offspring of HAmCq^G0^). Our results showed that besides *CYP9M10*, the expression of which was developmentally regulated and specifically overexpressed in the larval stage (4^th^ larval instar) compared with the adults, the expression of the other three P450 genes, *CYP6AA7*, *CYP9J34*, *CYP9J40,* was at similar levels in the larval and adult stages (Fig. 1). Significant differences in the expression of four P450 genes in the larval stage were identified among susceptible S-Lab, HAmCq^G0^ and HAmCq^G8^ mosquito populations (Fig. 1). The expression of *CYP6AA7* was overexpressed (∼2-fold) in the 4^th^ instar of HAmCq^G0^ compared with susceptible S-Lab mosquitoes, increasing to ∼5-fold in HAmCq^G8^ after permethrin selection (Fig. 1A). A similar expression pattern was also found for *CYP9M10*; the expression of the gene was ∼4-fold higher in the 4^th^ instar of HAmCq^G0^ compared with S-Lab, increasing to ∼11-fold in HAmCq^G8^ (Fig. 1D). Although the expression of *CYP9J34* in HAmCq^G0^ was similar to that in the S-Lab strain, the expression was significantly increased after permethrin selection (Fig. 1B). The correlation of the gene expression with the levels of resistance developed following permethrin selection suggests the importance of *CYP6AA7, CYP9M10* and *CYP9J34* in permethrin resistance in *Culex* mosquitoes.

Comparison of the gene expression among these four P450 genes in the adult stage of mosquitoes revealed that among the three P450 genes *CYP6AA7*, *CYP9J34*, *CYP9J40,* whose expression levels were similar for the larval and adult stages, only *CYP6AA7* showed a significant overexpression in HAmCq^G8^ mosquitoes following permethrin selection (∼3.5-fold, Fig. 1A). No significant difference was found in the expression of *CYP9J34* and *CYP9J40* at the adult stage among susceptible S-Lab, HAmCq^G0^ and highly resistant HAmCq^G8^ mosquitoes (Figs. 1B and 1C). These results suggest that *CYP9J34* and *CYP9J40* play no role in the development of resistance in adult HAmCq mosquitoes. These results further suggest that different mechanisms and/or P450 genes may be involved in the response to insecticide pressure for different developmental stages of mosquitoes and different populations of mosquitoes [27].

### Tissue specific overexpression of *CYP6AA7* in resistant and susceptible mosquitoes

**Figure 2:**
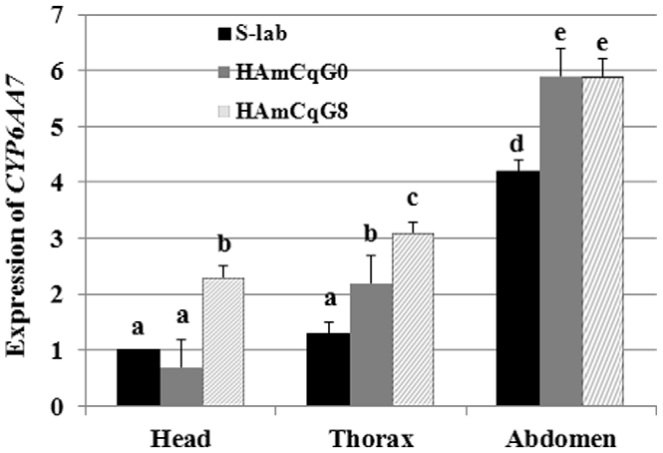
Expression of *CYP6AA7* in head, thorax, and abdomen tissue of 2–3 day-old female adult (without blood feeding) *Culex* mosquitoes. The relative level of gene expression shown along the Y axis represents the ratio of the gene expression in each tissue of each mosquito strain compared to that in the head of the susceptible S-lab strain ( = 1). The results are shown as the mean ± S.E. There was no significant difference (*P*≤0.05) in the levels of *CYP6AA7* expression among samples with the same alphabetic letter (i.e., a, b, or c).

Insect P450s may also vary as to the tissues where they are expressed in response to physiological and environmental stimulators. In insects, the midgut and fat body tissue are generally considered to be the primary detoxification organs where most insect detoxification P450s are expressed [28]. Nevertheless, other tissues, such as the brain [29] and nervous system [30] may also be important for P450 gene expression and response to insecticide resistance. Our study found that *CYP6AA7* was overexpressed not only in larvae of resistant HAmCq^G8^ mosquitoes, but also in adults of the same strain. To further characterize whether the overexpression of *CYP6AA7* is detoxification tissue specific, RNAs from the head, thorax, and abdomen of 2–3 day-old female adults (without blood feeding) of S-Lab, HAmCq^G0^ and HAmCq^G8^ mosquitoes were subjected to qRT-PCR analyses. Comparison of the levels of *CYP6AA7* expression among the three tissues indicated that it was lower in the head, increased in the thorax tissue and reached its highest concentration in the abdomen tissue of all three mosquito strains (Fig. 2). As midgut and most fat body components are located in the abdomen of insects and are known to be of primary importance in detoxification-related functions, the relatively high levels of *CYP6AA7* in the abdomens of all three mosquito strains suggest the importance of the gene in the detoxification of insecticides in mosquitoes. However, because midgut and fat body tissues are not exclusively found in the abdomen, further dissection of detoxification-related tissue (such as midgut and fat body) is needed to pinpoint the precise location for the overexpression of *CYP6AA7*. Significant overexpression was particularly evident in HAmCq^G8^ population in all three types of tissue (Fig. 2) and was closely correlated with each strain's level of insecticide resistance. The HAmCq^G0^ strain, with a relatively lower level of resistance, showed no significant difference in the *CYP6AA7* expression in the head tissues compared with the susceptible S-Lab strain but increased expression (2-fold) of *CYP6AA7* in both the thorax and abdomen tissues (Fig. 2).

### Response of P450 genes to permethrin challenge in resistant and susceptible mosquitoes

It has been proposed that many chemical inducers act as substrates for the P450s that they induce and that the induction of the P450s by the substrates will, in turn, reduce the effects of the substrates by enhancing substrate metabolism [17]. We thus hypothesized that insecticide resistant mosquitoes may be uniquely resistant to insecticides due to their ability to mount an adequate cellular response, for example the ability to up-regulate their production of P450s, when challenged with insecticides. We therefore compared the inducibility of expression of the four P450 genes, *CYP6AA7*, *CYP9J34*, *CYP9J40* and *CYP9M10*, among susceptible S-Lab, intermediate resistant HAmCq^G0^, and highly resistant HAmCq^G8^ mosquitoes.

**Figure 3:**
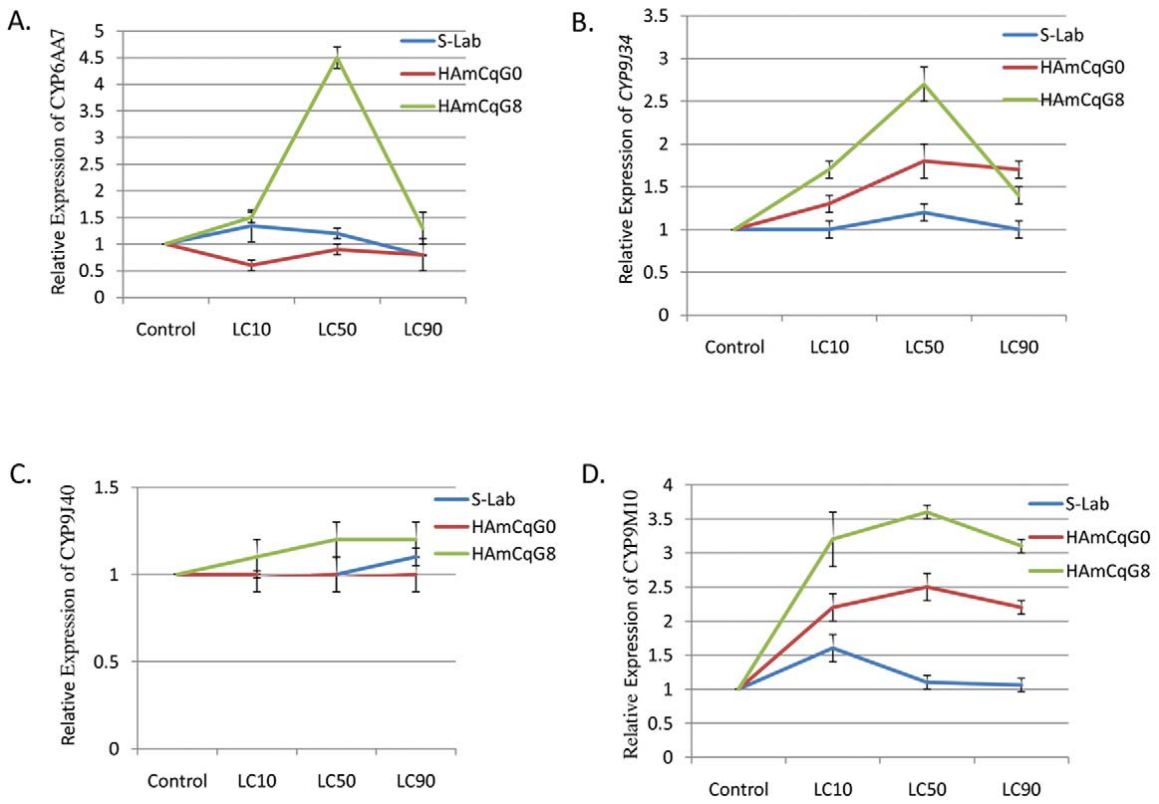
Dose-dependent induction of P450 expression following treatment with permethrin. The expression of *CYP6AA7*, *CYP9J34*, *CYP9J40*, and *CYP9M10* in late 3^rd^ instar larval mosquitoes *Cx. quinquefasciatus* in each of the mosquito populations 24 h after permethrin treatment with a corresponding concentration range of LC_10_, LC_50_, and LC_90_ (Table 1) was analyzed by qRT-PCR as described in Section 2.4, Materials and Methods. The relative level of gene expression shown along the Y axis represents the ratio of the gene expression in each treatment compared with that in acetone treated control mosquitoes. The experiments were repeated three times. The results are shown as the mean ± S.E.

**Figure 4:**
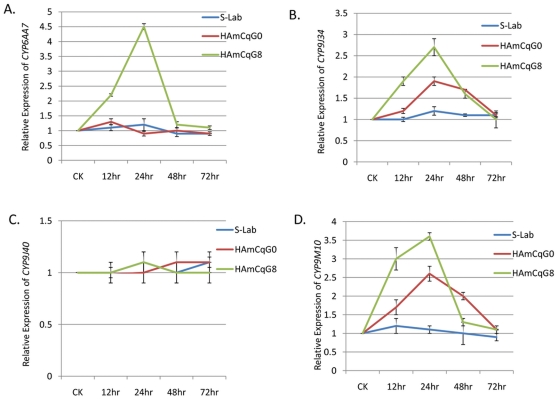
The duration of the gene expression following permethrin treatment at a concentration of LC_50_. The expression of *CYP6AA7*, *CYP9J34*, *CYP9J40*, and *CYP9M10* in *Cx. quinquefasciatus* following treatment with permethrin at the respective LC_50_ concentrations (0.007ppm, 0.07ppm, and 20ppm for the S-Lab, HAmCq^G0^, and HAmCq^G8^ strains, respectively) were analyzed 12, 24, 48, and 72h after the permethrin treatment. The relative level of gene expression shown along the Y axis represents the ratio of the gene expression in each treatment in comparison with that in acetone treated control mosquitoes. The experiments were repeated three times. The results are shown as the mean ± S.E.

To examine the effect of permethrin on induction of the four P450 genes, we measured the expression of the genes in late 3^rd^ instar larval mosquitoes challenged with permethrin at corresponding dose ranges (LC_10_, LC_50_, and LC_90_ for each strain) for various durations (Table 1, see Section 2.2 in Materials and Methods). Our preliminary results showed that although no significant induction was detected in the susceptible S-Lab mosquitoes for the dose range and time intervals tested (data not shown), permethrin induced three P450 genes in resistant HAmCq^G8^ mosquitoes with varying levels in a clear concentration dose- and time-dependent manner. Based on these data, a permethrin concentration of LC_50_ for each mosquito strain and a time interval of 24 h were chosen for the further induction studies (Figs 3 and 4) and the expression of four P450 genes in response to permethrin challenge in each of three mosquito populations was characterized. The duration of the P450 gene expression following permethrin treatment at the LC_50_ concentration and the expression of the genes 24 h after permethrin treatment over a concentration range of LC_10_, LC_50_, and LC_90_ were investigated. No significant induction in the expression of *CYP6AA7* was detected in susceptible S-Lab and HAmCq^G0^ that had been treated with either acetone alone (control) or with any of the three concentrations of permethrin solution in acetone at 24 h after treatment (Fig. 3A). However, in the HAmCq^G8^ strain, an initial induction of *CYP6AA7* (∼1.5-fold) was found in mosquitoes that had been treated with the LC_10_ of permethrin and a marked induction (∼4.5-fold) in the mosquitoes treated with the permethrin at a concentration of LC_50_. No significant induction was detected in the mosquitoes with a permethrin concentration of LC_90_. Although no induction of *CYP9J34* was detected in the susceptible S-Lab strain, elevated levels of *CYP9J34* expression were detected in HAmCq^G0^ mosquitoes treated with permethrin compared with the corresponding no-permethrin treated control. The levels of *CYP9J34* RNA in HAmCq^G0^ were readily induced by LC_10_ permethrin concentration, induced to a maximum (∼1.7-fold) by LC_50_ permethrin concentration, with no further significant induction up to LC_90_ of permethrin concentration (Fig. 3B). Nevertheless, significant induction of *CYP9J34* was more evident in the HAmCq^G8^ strain than in their parental HAmCq^G0^, with an induction peak of ∼2.7-fold at a permethrin concentration of LC_50_ (Fig. 3B). A similar induction pattern was also found for *CYP9M10* RNA (Fig. 3D) in HAmCq^G0^ and HAmCq^G8^. However, no significant induction of *CYP9J40* was identified in any of the three mosquito strains tested (Fig. 3C). The significant induction of the P450 genes only in the field resistant and/or permethrin selected highly resistant mosquito strains suggests their importance in the resistant mosquitoes' response to permethrin treatment.

Examining the durations of P450 gene induction with LC_50_ permethrin concentration treatment revealed no significant induction in the expression of *CYP6AA7* in susceptible S-Lab and HAmCq^G0^ at any time after the treatment (Fig. 4A). However, in the HAmCq^G8^ strain, the initial induction (∼2.5-fold) was found 12 h after LC_50_ permethrin concentration (20 ppm) treatment, reaching a peak at 24 h after permethrin treatment with an induction level of 4.5-fold, and declining dramatically 48 h after treatment (Fig. 4A). While we did not detect the induction of either *CYP9J34* or *CYP9M10* in the susceptible S-Lab strain at any time after the mosquitoes were treated with LC_50_ permethrin concentration (0.007 ppm), elevated levels of *CYP9J34* and *CYP9M10* expression were detected in HAmCq^G0^ mosquitoes treated with permethrin at the LC_50_ concentration (0.07 ppm) compared with their no-permethrin treated controls (Figs. 4B and 4D). The induction for both *CYP9J34* and *CYP9M10* in HAmCq^G0^ reached a maximum (∼1.9- or 2.3-fold, respectively) for both genes 24 h after permethrin LC_50_ concentration treatment. The induction levels of both genes then declined by 48 h after treatment, with no significant induction (*p≤0.05) detected after 72 h treatment compared with untreated or acetone treated mosquitoes (Figs. 4B and 4D). Similarly, the induction of both *CYP9J34* and *CYP9M10* in HAmCq^G8^ reached a maximum (∼2.7- or 3.7-fold, respectively) for both genes at 24 h after permethrin LC_50_ concentration treatment, declining after 48 h treatment (Figs. 4B and 4D). No significant induction of *CYP9J40* was identified in any of the three mosquito strains at any time after the mosquitoes had been treated by permethrin at their corresponding LC_50_ concentrations (Fig. 4C). The significant induction of the P450 genes only in field resistant and/or permethrin selected highly resistant mosquito strains suggests their importance in the response to permethrin treatment of resistant mosquitoes.

## Discussion

In many cases, increased levels of P450 gene expression (i.e., the overexpression of P450 genes) are known to result in increased levels of total P450s and the P450 activities that are responsible for insecticide resistance [3], [7], [11], [16], [31], [32]. Both constitutively increased expression (overexpression) and induction of P450s are thought to be responsible for increased levels of detoxification of insecticides [1]. Multiple P450 genes that are induced in insects in response to host plant allelochemicals or secondary products have been extensively studied and are fairly well documented in terms of their function in the adaptation of insects in “animal-plant warfare” [33] and in the co-evolution of insects and plants [34]. In contrast, P450 gene induction in response to insecticide resistance is less well understood.

Our previous research has indicated that resistance in HAmCq, the *Cx. quinquefasciatus* mosquito strain used in this research, could be partially suppressed by piperonyl butoxide (PBO), an inhibitor of cytochrome P450s [35]. Further study identified several P450 EST sequences that were overexpressed in resistant HAmCq mosquitoes [19]. Nevertheless, until now no individual P450 genes have been isolated and characterized in the HAmCq mosquitoes as being responsible for resistance. In the current study, we isolated and sequenced four P450 cDNAs, *CYP6AA7*, *CYP9J40*, *CYP9J34*, and *CYP9M10*, from mosquitoes *Cx. quinquefasciatus*. Although three of them, *CYP6AA7*, *CYP9J40*, and *CYP9J34*, have not previously been reported in *Culex* mosquitoes in terms of insecticide resistance, the overexpression of *CYP9M10* has been reported in a resistant *Culex* mosquito strain in Japan [26], and has been further suggested to be linked with pyrethroid resistance in *Culex* mosquito [36], [37]. The coincidence of the overexpression of *CYP9M10* in resistant *Culex* mosquito populations in Japan and US may strongly suggest a common feature of this P450 gene in pyrethroid resistance in mosquitoes, *Culex quinquefasciatus*.

In this study, both the constitutive overexpression of these P450 genes and the induction of the P450 genes in response to a challenge with insecticides in resistant *Cx. quinquefasciatus* were characterized. Clear correlations were found between the levels of P450 gene expression or induction and the levels of permethrin resistance or susceptibility among the susceptible S-Lab strain, low resistant strain HAmCq^G0^, and the highly resistant HAmCq^G8^ strain. Because insecticide resistance is generally assumed to be a pre-adaptive phenomenon, where prior to insecticide exposure rare individuals carrying an altered (varied) genome already exist thus allowing the survival of those carrying the genetic variance after insecticide selection, we expected that the number of individuals carrying the resistance genes or alleles should increase in a population following selection and become predominate in the population. The approach adopted for this study, which compared P450 gene expression and induction among different mosquito populations and between the parental field population, HAmCq^G0^, and its permethrin selected offspring, HAmCq^G8^, for different levels of insecticide resistance highlighted the importance of P450 genes in resistance by detecting changes in their expression within each population following permethrin selection. We restricted the induction response to permethrin treatment because it is the insecticide that these mosquitoes are resistant to. We found that the overexpression levels of four P450 genes (*CYP6AA7*, *CYP9J40*, *CYP9J34*, and *CYP9M10*) in all three mosquito populations were closely correlated to their levels of resistance and were higher in , HAmCq^G8^, compared to the parent strain HAmCq^G0^. Furthermore, we also found that the induction levels of *CYP6AA7*, *CYP9J34*, and *CYP9M10*, but not *CYP9J40*, in the mosquito populations correlated with their levels of resistance and were again higher in HAmCq^G8^ compared to HAmCq^G0^. Our study strongly indicates that the overexpressed P450 genes are more strongly induced when the mosquitoes are exposed to insecticides, which, in turn, increase the overall expression levels of multiple P450 genes in resistant mosquitoes. We also observed that P450 gene induction in mosquitoes follows a resistance-specific pattern; similar results have been reported in *Drosophila melanogaster* [38], where the expression of *CYP6g1* and *CYP12d1* were induced in the DDT resistant strains post-exposure to DDT. Recent studies by Zhu et al. [11], [16] indicated that several P450 genes were up-regulated in insecticide resistant house flies through a similar induction mechanism. Taken together, these studies strongly suggest a common mechanism for P450 induction in response to detoxification-mediated insecticide resistance in a number of different insect species.

### Conclusions

This study provides direct evidence that four P450 genes, *CYP6AA7*, *CYP9J40*, *CYP9J34*, and *CYP9M10*, are up-regulated in insecticide resistant mosquitoes through constitutive overexpression and/or induction mechanisms. As this was found only in resistant mosquitoes, and was markedly higher in the permethrin selected highly resistant mosquitoes, the results strongly suggests the functional importance of these four P450 genes in the increased detoxification of insecticides in resistant *Culex* mosquitoes. Both P450 induction and constitutive overexpression may be co-responsible for detoxification of insecticides, evolutionary insecticide selection, and the ability of insects to adapt to changing environments.
